# A systematic review and meta-analysis of the diagnostic value of circulating microRNA-17-5p in patients with non-small cell lung cancer

**DOI:** 10.1097/MD.0000000000033070

**Published:** 2023-02-22

**Authors:** Juntao Hao, Zengqiang Shen

**Affiliations:** a Department of Thoracic Surgery, Shanxi Provincial People’s Hospital, Taiyuan, China.

**Keywords:** 17, 5p, analysis, diagnostic value, meta, microRNA, NSCLC

## Abstract

**Background:**

nonSmall Cell Lung Cancer (NSCLC) is the most common type of lung cancer with high morbidity and mortality rates. Studies have shown that miR-17-5p levels are significantly increased in the circulating blood of NSCLC patients. This meta-analysis aimed to investigate the diagnostic value of miR-17-5p in NSCLC in China.

**Methods:**

A literature search was conducted for studies on the correlation between miR-25 and the diagnosis of NSCLC until October 2022 using English and Chinese databases. The Quality Assessment of Diagnostic Accuracy Studies (QUADAS-2) was adopted to evaluate the quality of studies in the literature. Numerical values for sensitivity and specificity were obtained from false negative (FN), false positive (FP), true negative (TN), and true positive (TP) rates, presented alongside graphical representations with boxes marking the values and horizontal lines showing the confidence intervals. Summary Receiver Operating Characteristic (SROC) curves were applied to assess the performance of the diagnostic tests. The data were processed using RevMan 5.3.

**Results:**

Three studies (208 cases of NSCLC patients and 198 healthy controls) met our evaluation criteria. The sensitivity was 0.70 to 0.75, and the specificity value was 0.82 to 0.83. The Area Under the Curve (AUC) from the SROC curves was > 80%; therefore, it was classified as a good category.

**Conclusion:**

Our meta-analysis shows that miR-17-5p can be used for the diagnosis of NSCLC and may serve as a biomarker for the detection of early NSCLC in the Chinese population.

## 1. Introduction

Lung cancer is the most common malignant tumor worldwide.^[1]^ The mortality rate from lung cancer, especially in China, is on the rise.^[2]^ According to the latest cancer epidemiology data in China reported by the National Cancer Research Center of China in 2016, lung cancer is the leading cause of cancer-related death in terms of both morbidity and mortality, which indicates that lung cancer has become the largest burden on public health in China. nonsmall cell lung cancer (NSCLC) is the main type of lung cancer, accounting for up to 85% of all cases.^[3]^ The overall cure and survival rates of NSCLC remain low, especially for patients with metastasis, with a 5-year survival rate of less than 5%.^[4]^ Owing to the lack of specific symptoms, patients are often not diagnosed until the late stage and miss the best treatment opportunities, such as surgery. Therefore, to provide effective treatment strategies and improve the survival rate of patients with NSCLC, it is necessary to explore effective molecular markers for the early diagnosis and prognosis of NSCLC.

MicroRNAs (miRNAs) are a class of short endogenous noncoding RNAs (around 22 nucleotides on average) that play critical roles in cancer development and progression.^[5,6]^ They regulate oncogenic and/or tumor suppressor genes by binding to seed sequences within the 3′ translation region (UTR) of the target mRNA, ultimately leading to degradation of the target mRNA or blocking of protein translation.^[7]^ The need for noninvasive NSCLC diagnosis has increased substantially in recent years, as new noninvasive biomarker miRNAs have been used for the genomic diagnosis of NSCLC. MiR-17-5p is an important member of the miR-17-92 cluster.^[8]^ The normal miR-17-92 cluster is necessary for normal lung development, and its altered expression has been reported in various lung diseases such as NSCLC.^[9,10]^ It has been reported that the serum level of miR-17-5p in NSCLC patients is significantly higher than that in healthy people.^[11]^

These results indicate that miR-17-5p has a potentially important clinical value in predicting the prognosis of NSCLC patients. Therefore, this meta-analysis aimed to explore the value of miR-17-5p in predicting the prognosis of NSCLC *via* comprehensive analysis of data from multiple studies.

## 2. Materials and methods

### 2.1. Study search

A systematic review was conducted according to the methods and recommendations of the PRISMA (Preferred Reporting Items for Systematic Reviews and Meta-Analyses) extension statement. We identified studies published in English and Chinese to evaluate the diagnostic value of miR-17-5p in NSCLC. English (PubMed, Google Scholar, Cochrane Library, and Clinical Trials) and Chinese (CNKI, Cqvip, WANFANG data, and Baidu scholar) databases were searched using the following retrieval strategies: (microRNA-17-5p OR miRNA-17-5p OR microRNA17-5p, OR miR-17-5p OR miRNA17-5p OR hsa-miR-17-5p) AND (lung tumor OR NSCLC OR NSCLC OR lung neoplasms OR lung cancer). The reference lists of included studies were reviewed to identify other potentially eligible studies.

### 2.2. Exclusion criteria

The selected publications were independently evaluated by 2 reviewers based on the established inclusion criteria. These publications were selected based on the information provided in the title and/or abstract as well as their full text, and the language was limited to English and Chinese. Firstly, these publications were studies on the expression of miR-17-5p and the diagnostic value of NSCLC. Moreover, all subjects were suspected patients with diagnosed NSCLC. Additionally, all patients were diagnosed with NSCLC according to the clinical gold standard. Furthermore, the research needs to provide enough information to build 2 × 2 contingency table, that is, false and true positives and negatives were provided.

The following studies were excluded from this systematic review and meta-analysis: nonresearch-based publications, such as letters, case reports, reviews, conference summaries, and animal or laboratory studies; publications without reports of no receiver operating characteristic curve and the specific values of sensitivity and specificity; and publications containing unclear diagnostic criteria or unconventional diagnostic tools.

### 2.3. Data extraction and quality assessment

The corresponding data were extracted from studies that met the inclusion criteria and contained the following information: first author, year of publication, country, sample size (including numbers of experimental groups and control groups), sample types, main experimental methods, the gold standard, the summary receiver operating characteristic (SROC) curve, and the area under the curve (AUC). Indicators such as sensitivity and specificity, numbers of true positive (TP), false positive (FP), false negative (FN), and true negative (TN) were recorded directly or calculated indirectly according to the original data of the enrolled studies. The quality of the enrolled studies was assessed using the Cochrane Centre Quality Assessment of Diagnostic Accuracy Studies (QUADAS-2). To avoid bias, when the evaluations of the 2 investigators were inconsistent, a third investigator (GB) was invited for further discussions to eliminate the differences. Assessment of the included studies was conducted in a single-blind manner.

### 2.4. Statistical analysis

Literature quality was evaluated using the QUADAS-2 tool of RevMan 5.3 (Cochrane Collaboration, Oxford, UK). The meta-analysis was performed using Review Manager 5.3. Forest plots were used to summarize the estimates with 95% confidence intervals. The random-effects model was used to calculate pooled sensitivity, specificity, positive predictive value, and negative predictive value. The SROC curve was plotted, and the AUC of the SROC was then calculated. A rough guide for classifying the accuracy of a diagnostic test was based on the AUC. The criteria for AUC classification are 90% to 100% (excellent), 80% to 90% (good), 70% to 80% (fair), 60% to 70% (poor), and 50% to 60% (failure).^[12]^

## 3. Results

### 3.1. Description of studies

**Figure 1.**
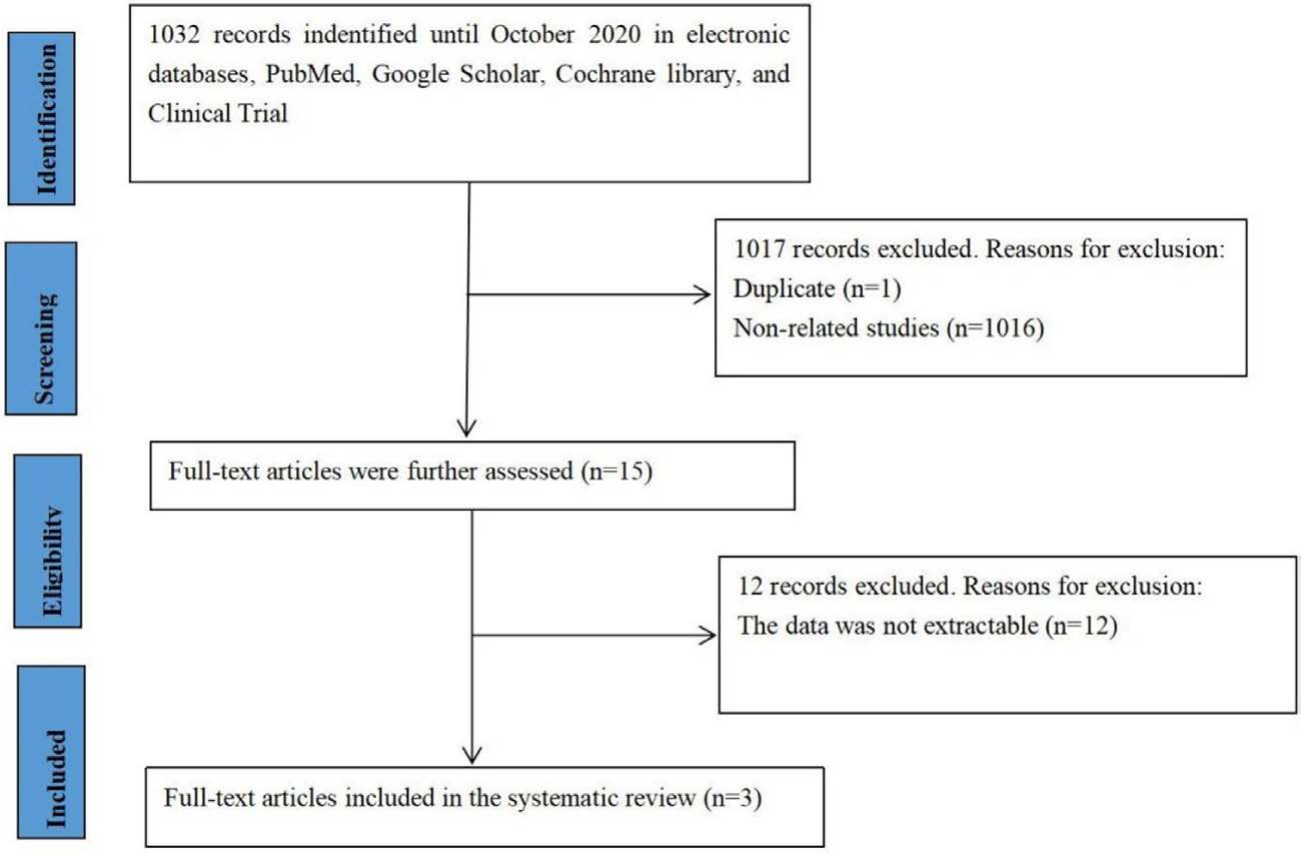
Flow chart of the study selection process.

Based on the search strategies of the databases, 1032 Chinese or English articles were identified. Fifteen full-text articles were selected, and duplicate citations and studies not relevant to the current meta-analysis were removed. After excluding 12 articles with incomplete data, 3 met the inclusion criteria and were included in the systematic review (Fig. 1). Among the 3 articles, 2 were in Chinese and 1 was in English (Table 1). A total of 208 patients with NSCLC and 198 healthy controls were included in the 3 studies. The basic characteristics of the included literature are shown in Table 1.

**Table 1:** Characteristics of studies included in the present meta-analysis.

Reference	Yr	Country	Patients/controls	Detection matrix	Method	TP	FP	FN	TN	Sensitivity (95% CI)	Specificity (95% CI)
Jian Liu^[13]^	2021	China	60/60	Serum	qRT-PCR	42	11	18	49	46 (36, 57)	62 (42, 79)
Ping Wei^[14]^	2022	China	48/48	Serum	qRT-PCR	36	8	12	40	47 (36, 59)	60 (36, 81)
Yi Zhang^[11]^	2019	China	100/90	Serum	qRT-PCR	70	16	30	74	49 (40, 57)	43 (24, 63)

CI = confidence intervals, FN = false negative, FP = false positive, TN = true negative, TP = numbers of true positive.

### 3.2. Publication bias

**Figure 2.**
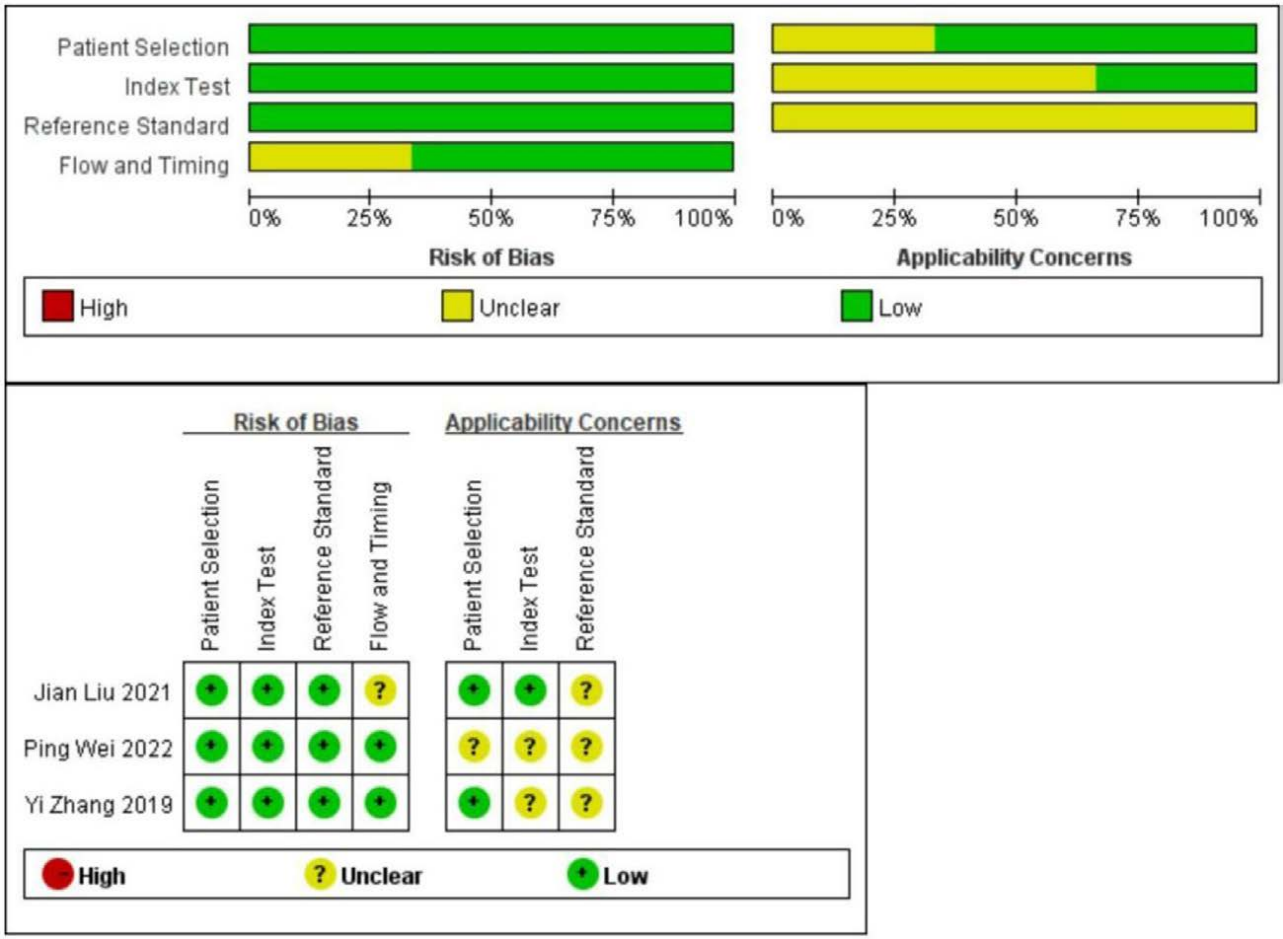
Quality of selected studies according to QUADAS-2 guidelines. QUADAS-2 = the quality assessment of diagnostic accuracy studies.

The QUADAS-2 tool was used to assess the quality of the included studies (Fig. 2). We evaluated the quality of the diagnostic studies based on the QUADAS-2 criteria in 4 key areas: patient selection, index testing, reference criteria, flow and timing, judgment bias, and applicability. Each area was assessed in terms of risk of bias, and the first 3 areas were assessed in terms of suitability. The outcome of the assessments can be “yes,” “no,” or “unclear” for each item. A “yes” implies a lower risk of bias, while a “no” or “unclear” outcome implies the opposite (Fig. 2).

### 3.3. Meta-analysis

**Figure 3.**
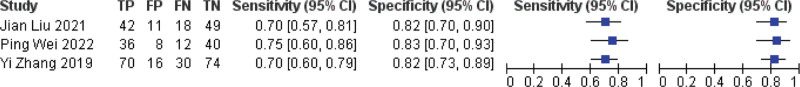
Forest plot of miR-17-5p for the diagnosis of NSCLC. NSCLC = nonsmall cell lung cancer.

**Figure 4.**
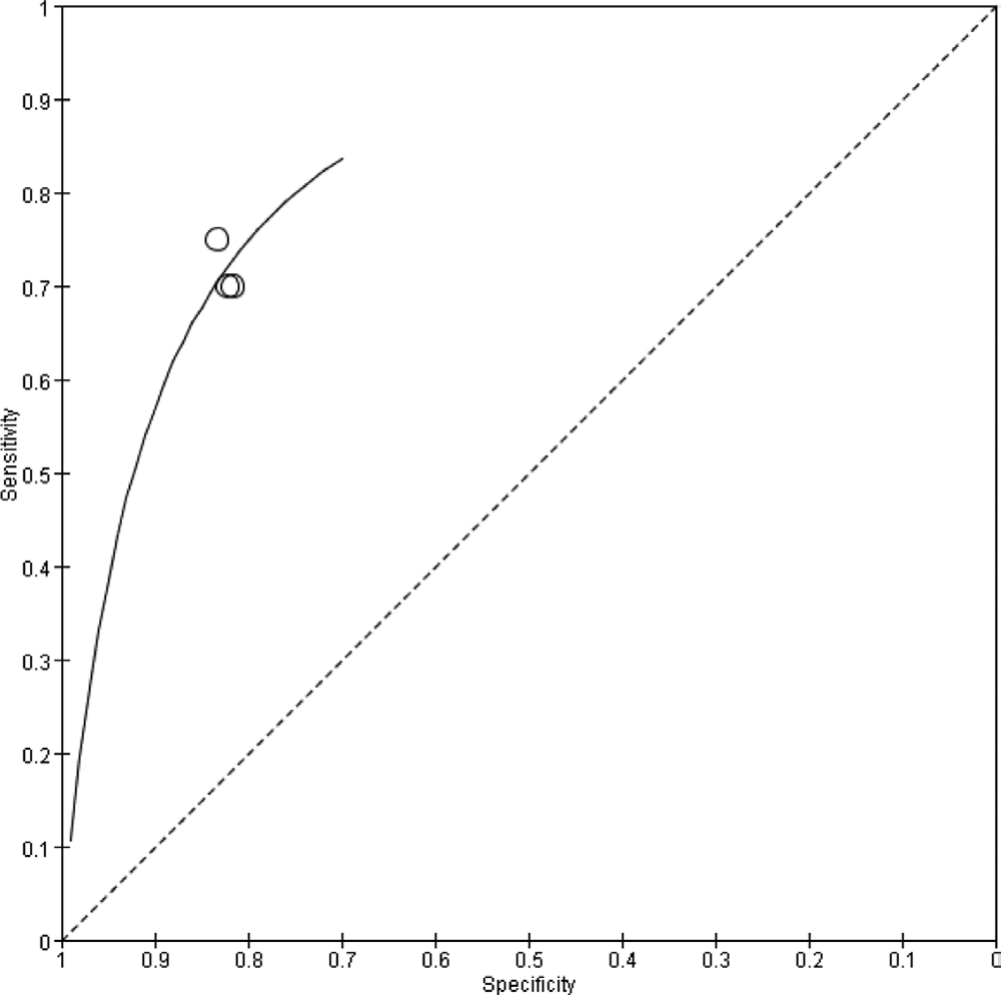
SROC plot for miR-17-5p in the diagnosis of NSCLC. NSCLC = nonsmall cell lung cancer, SROC = summary receiver operating characteristic.

The results of diagnostic accuracy analysis showed that the sensitivity value of miR-17-5p was between 0.70 and 0.75, while the specificity value was between 0.82 and 0.83 (Fig. 3). An SROC plot based on the results of this meta-analysis was then constructed, and the pooled AUC of SROC was found to be >80% (Fig. 4).

## 4. Discussion

More than 1000 mature miRNAs have been identified in the human genome. Although miRNAs make up only 1% to 3% of the genome, they regulate more than 30% of protein expression in the body.^[15]^ The expression of some miRNAs has been found to be significantly different between tumor cells and corresponding normal cells, indicating that miRNAs may play a crucial role in the development of human tumors.^[16]^ The discovery of miRNAs, especially serum miRNAs, has opened up a new avenue for early diagnosis of cancer. Studies have found that abnormal expression of miRNAs exists not only in lung cancer tissues but also in blood circulation, and miRNAs can be stably present in serum.^[16,17]^

The normal miR-17-92 cluster is expressed in many human cancers and does not inhibit cell growth; therefore, it is often called onco-miR-1.^[18]^ Phosphate and tension protein homologue is recognized as 1 of miR-17-92 cluster validated targets.^[19]^ Overexpression of the miR-17-92 cluster and subsequent inhibition of phosphate and tension protein homologue expression have been observed in a variety of cancer cells.^[20]^ Furthermore, miR-17-5p is the most important member of the miR-17-92 cluster and an important regulator of basic cellular processes, such as cell proliferation, autophagy, and apoptosis.^[21]^ Furthermore, many cancer types are associated with elevated miR-17-5p expression.^[22,23]^ It has been reported that the relative expression level of miR-17-5p in the serum of patients with NSCLC is higher than that in healthy controls. In addition, serum miR-17-5p levels were negatively correlated with survival in lung cancer patients.^[24]^

This study represents the first meta-analysis of the diagnostic utility of miR-17-5p in patients with NSCLC. Before the analysis, we conducted a methodological quality assessment based on the QUADAS-2 criteria. All 3 of the included studies effectively distinguished patients with NSCLC from healthy individuals. For the quality assessment of patient sampling, we found that the description of patient sampling was clear in all the 3 included studies. In the flow and time sections of the quality assessment, we found no reference to the allocation interval between miR-17-5p detection and gold standard diagnosis. However, because this study was conducted only in patients with lung cancer, the length of the interval did not change the risk of lung cancer. Therefore, we considered the miR-17-5p detection time and gold standard time interval as low-risk events.

The 3 included studies comprise 208 patients with NSCLC and 198 healthy controls. By meta-analysis, we found that the pooled AUC of SROC was > 80% in the diagnosis of NSCLC, and the sensitivity value of miR-17-5p was between 0.70 and 0.75, while the specificity value was between 0.82 to 0.83 in the diagnosis of NSCLC. Our data suggests that miR-17-5p may be a potential diagnostic marker for NSCLC. In addition, it has been suggested that miR-17-5p can be combined with other tumor markers to improve its diagnostic accuracy for NSCLC. It has been reported that the area under the receiver operating characteristic curve for miR-483-5p, miR-193a-3p, miR-25, miR-214, and miR-7 combined in the diagnosis of NSCLC can be as high as 0.976, which suggests the need to continue evaluating, exploring, and combining new tumor markers to improve the diagnostic efficiency of NSCLC.

A limitation of this study is that, in terms of diagnostic accuracy, only 3 studies met the inclusion criteria, and the insufficient sample size may have affected the overall estimation. In addition, the population included in this study was relatively narrow, all of which were Chinese; therefore, there may be some limitations in the generalization of the conclusions. Moreover, only papers published in English or Chinese were included in the included literature, and papers in other languages were excluded, leading to inevitable errors.

Nonetheless, in conclusion, our analysis shows that miR-17-5p is a relatively promising and effective biomarker for differentiating patients with NSCLC from healthy individuals. It can be a novel noninvasive diagnostic biomarker for NSCLC diagnosis with acceptable sensitivity and specificity and good AUC of SROC. However, owing to the limitations of this study, high quality, large sample sizes from multiple centers are still needed for further validation.

## Author contributions

**Conceptualization:** Juntao Hao.

**Data curation:** Juntao Hao, Zengqiang Shen.

**Methodology:** Juntao Hao, Zengqiang Shen.

**Software:** Juntao Hao, Zengqiang Shen.

**Validation:** Zengqiang Shen.

**Writing – original draft:** Juntao Hao.

**Writing – review & editing:** Juntao Hao.
